# New Insights into the Biological and Clinical Aspects of Merkel Cell Carcinoma

**DOI:** 10.3390/cancers13092259

**Published:** 2021-05-08

**Authors:** Virve Koljonen, Weng-Onn Lui, Jürgen C. Becker

**Affiliations:** 1 Department of Plastic Surgery, University of Helsinki and Helsinki University Hospital, 00029 Helsinki, Finland; 2 Department of Oncology-Pathology, Karolinska Institutet, BioClinicum, Karolinska University Hospital, 171 64 Solna, Sweden; 3 Translational Skin Cancer Research, German Cancer Consortium (DKTK), 45151 Essen, Germany; 4 German Cancer Research Center (DKFZ), 69120 Heidelberg, Germany

The Special Issue in *Cancers*, “The Biological and Clinical Aspects of Merkel Cell Carcinoma”, walks the avid reader through the interesting and sometimes even mysterious facets of Merkel cell carcinoma (MCC), starting at its carcinogenesis to also cover innovative treatment options.

The groundworks for MCC and its causative agent Merkel cell polyomavirus (MCPyV) are laid in an exhaustive review by Pietropaolo et al. [1]. They provide a comprehensive review of the current knowledge and spell out the undisputed role of MCPyV in oncogenesis in viral-associated MCC. Further and current evidence for the MCPyV oncogenic functions is provided by Spurgeon et al. [2]. With murine skin cancer model they show that MCPyV T antigens function in tumor promotion but not in initiation. Leaving one of the most enigmatic questions in MCC, open and thus vacant for future research.

To date, the effective treatment options for advanced MCC are still limited. In this issue, an interesting article by Sarma et al. [3] tested the effect of artesunate, an anti-malaria compound listed in the World Health Organization essential medicines [4], on MCC. They show that artesunate represses MCPyV T-antigen expression and inhibits cell growth in vitro and in vivo, suggesting its potential treatment for MCC. Fan et al. [5] concede that miR-375 is unlikely an intracellular oncogene in MCC cells and thus may rather serve for intercellular communication; indeed they subsequently published that miR-375 is functional in polarizing cancer associated fibroblasts [6]. Kervarrec et al. [7] take on the complexity of cell of origin in MCC, in which they conclude that MCPyV T antigens contribute to the acquisition of Merkel cell-like phenotype in epithelial cells.

Turning to clinical patient care, Sahi et al. [8], portray a grim picture on real life experience on the treatment of MCC patients. Although limited to Finland, it is presumed that similar situation is a common and worldwide problem, not only with MCC patients, but rather in all patients with rare cancers. Björn Andtback et al. [9] review on their past experience on adjuvant radiation therapy in MCC, strengthening the previous notion that female MCC patients, regardless of MCPyV status, actually do better compared with their male counterparts. The third clinical paper by Naseri et al. [10] summarize the consensus treatment recommendations by the Danish MCC expert group. A second paper by Naseri et al. [11] described prognostic markers which hold the potential to stratify MCC patients for different treatment regimens.

Rare cancers pose a major challenge to the medical and scientific community [12]. Due to low patient numbers and thus limited market potentials, development and testing innovative therapeutic intervention is not prioritized by the pharmacological industry. Indeed, less common cancer subtypes and rare cancers are frequently only included in basket trials among several different entities, which may leave less attention to differing responses. Consequently, the lack of good and well-established treatments and clinical practices produces varying treatments and varying results. Furthermore, patients with rare cancers are worse off than other cancer patients [12], because these are often not diagnosed in a timely or correct manner. False diagnoses are more often than other patients or the correct diagnosis is delayed; in either case allowing the disease to progress before adequate therapy is initiated; thus, response to treatment is not as good as it could be. Getting peer support is often overwhelming.

It is often thought that the rarity of a specific cancer, such as MCC, causes patients to being “under-diagnosed” and to receive “under-treatment”, which is both unfortunately true. Published data on rare cancer are frequently based on a few patient cases or minor series with inadequate reporting [13], results that are not generalizable and it is difficult to establish a cause-and-effect relationship [14]. For example, due to the reporting bias for “successfully treated cases”, chemotherapy for MCC may have been advocated longer that it was reasonable [15]. Both clinicians and journal editors should keep this notions in mind [14].

Note added in Proof:

After preparation of this editorial, two additional manuscripts were accepted. Horny et al. [16] revealed mutational landscape of virus-positive and –negative MCC cell lines that is comparable to tumor samples, suggesting their utility as preclinical models for functional studies. Hill et al. [17] suggested three subgroups of MCC based on genomic copy number variants.

## References

[1] Pietropaolo V., Prezioso C., Moens U. (2020). Merkel cell polyomavirus and Merkel cell carcinoma. Cancers.

[2] Spurgeon M.E., Liem A., Buehler D., Cheng J., DeCaprio J.A., Lambert P.F. (2021). The Merkel Cell Polyomavirus T Antigens Function as Tumor Promoters in Murine Skin. Cancers.

[3] Sarma B., Willmes C., Angerer L., Adam C., Becker J.C., Kervarrec T., Schrama D., Houben R. (2020). Artesunate Affects T Antigen expression and survival of virus-positive Merkel cell carcinoma. Cancers.

[4] WHO (2019). World Health Organization Model List of Essential Medicines: 21st List 2019; World Health Organization. https://apps.who.int/iris/handle/10665/325771.

[5] Fan K., Zebisch A., Horny K., Schrama D., Becker J.C. (2020). Highly expressed miR-375 is not an intracellular oncogene in Merkel cell polyomavirus-associated Merkel cell carcinoma. Cancers.

[6] Fan K., Spassova I., Gravemeyer J., Ritter C., Horny K., Lange A., Gambichler T., Odum N., Schrama D., Schadendorf D. (2020). Merkel cell carcinoma-derived exosome-shuttle miR-375 induces fibroblast polarization by inhibition of RBPJ and p53. Oncogene.

[7] Kervarrec T., Samimi M., Hesbacher S., Berthon P., Wobser M., Sallot A., Sarma B., Schweinitzer S., Gandon T., Destrieux C. (2020). Merkel cell polyomavirus T antigens induce Merkel cell-like differentiation in GLI1-expressing epithelial cells. Cancers.

[8] Sahi H., Their J., Gissler M., Koljonen V. (2020). Merkel Cell Carcinoma Treatment in Finland in 1986–2016-A Real-World Data Study. Cancers.

[9] Björn Andtback H., Björnhagen-Säfwenberg V., Shi H., Lui W.-O., Masucci G.V., Villabona L. (2021). Sex Differences in Overall Survival and the Effect of Radiotherapy in Merkel Cell Carcinoma—A Retrospective Analysis of a Swedish Cohort. Cancers.

[10] Naseri S., Steiniche T., Ladekarl M., Bønnelykke-Behrndtz M.L., Hölmich L.R., Langer S.W., Venzo A., Tabaksblat E., Klausen S., Skaarup Larsen M. (2020). Management Recommendations for Merkel Cell Carcinoma—A Danish Perspective. Cancers.

[11] Naseri S., Steiniche T., Georgsen J.B., Thomsen R., Ladekarl M., Heje M., Damsgaard T.E., Bonnelykke-Behrndtz M.L. (2020). Tumor Ulceration, Reduced Infiltration of CD8-Lymphocytes, High Neutrophil-to-CD8-Lymphocyte Ratio and Absence of MC Virus are Negative Prognostic Markers for Patients with Merkel Cell Carcinoma. Cancers.

[12] Gatta G., Capocaccia R., Botta L., Mallone S., De Angelis R., Ardanaz E., Comber H., Dimitrova N., Leinonen M.K., Siesling S. (2017). Burden and centralised treatment in Europe of rare tumours: Results of RARECAREnet-a population-based study. Lancet Oncol..

[13] Kaszkin-Bettag M., Hildebrandt W. (2012). Case reports on cancer therapies: The urgent need to improve the reporting quality. Glob. Adv. Health Med..

[14] Nissen T., Wynn R. (2014). The clinical case report: A review of its merits and limitations. BMC Res. Notes.

[15] Nghiem P., Kaufman H.L., Bharmal M., Mahnke L., Phatak H., Becker J.C. (2017). Systematic literature review of efficacy, safety and tolerability outcomes of chemotherapy regimens in patients with metastatic Merkel cell carcinoma. Future Oncol..

[16] Horny K., Gerhardt P., Hebel-Cherouny A., Wülbeck C., Utikal J., Becker J.C. (2021). Mutational Landscape of Virus- and UV-Associated Merkel Cell Carcinoma Cell Lines Is Comparable to Tumor Tissue. Cancers.

[17] Hill N.T., Kim D., Busam K.J., Chu E.Y., Green C., Brownell I. (2021). Distinct Signatures of Genomic Copy Number Variants Define Subgroups of Merkel Cell Carcinoma Tumors. Cancers.

